# Structural Features Influencing the Bioactive Conformation of Angiotensin II and Angiotensin A: Relationship between Receptor Desensitization, Addiction, and the Blood–Brain Barrier

**DOI:** 10.3390/ijms25115779

**Published:** 2024-05-26

**Authors:** Graham J. Moore, Harry Ridway, Laura Kate Gadanec, Vasso Apostolopoulos, Anthony Zulli, Jordan Swiderski, Konstantinos Kelaidonis, Veroniki P. Vidali, Minos-Timotheos Matsoukas, Christos T. Chasapis, John M. Matsoukas

**Affiliations:** 1 Pepmetics Inc., 772 Murphy Place, Victoria, BC V8Y 3H4, Canada; mooregj@shaw.ca; 2 Department of Physiology and Pharmacology, Cumming School of Medicine, University of Calgary, Calgary, AB T2N 1N4, Canada; 3 Institute for Sustainable Industries and Liveable Cities, Victoria University, Melbourne, VIC 8001, Australia; ridgway@vtc.net; 4 Institute for Health and Sport, Immunology and Translational Research, Victoria University, Melbourne, VIC 3030, Australia; laura.gadanec@live.vu.edu.au (L.K.G.); vasso.aspostolopoulos@vu.edu.au (V.A.); anthony.zulli@vu.edu.au (A.Z.); jordan.swiderski@live.vu.edu.au (J.S.); 5 Immunology Program, Australian Institute for Musculoskeletal Science (AIMSS), Melbourne, VIC 3021, Australia; 6 NewDrug/NeoFar PC, Patras Science Park, 26504 Patras, Greece; k.kelaidonis@gmail.com; 7 Institute of Nanoscience and Nanotechnology, National Centre for Scientific Research “Demokritos”, 15341 Athens, Greece; v.vidali@inn.demokritos.gr; 8 Department of Biomedical Engineering, University of West Attica, 12243 Athens, Greece; minosmatsoukas@upatras.gr; 9 Institute of Chemical Biology, National Hellenic Research Foundation, 11635 Athens, Greece; cchasapis@eie.gr; 10 Department of Chemistry, University of Patras, 26504 Patras, Greece

**Keywords:** addiction, arginine, angiotensin II, bisartan, blood–brain barrier, conformation of angiotensin, coronavirus disease 2019, G-protein coupled receptor, receptor desensitization

## Abstract

The N-terminal portion of the octapeptide angiotensin II (DRVYIHPF; AngII), a vasopressor peptide that favorably binds to, and activates, AngII type 1 receptor (AT_1_R), has an important role in maintaining bioactive conformation. It involves all three charged groups, namely (i) the N-terminal amino group cation, (ii) the Asp sidechain anion and (iii) the Arg guanidino cation. Neutralization of any one of these three charged groups results in a substantial reduction (<5%) in bioactivity, implicating a specialized function for this cluster. In contrast, angiotensin A (ARVYIHPF; AngA) has reduced bioactivity at AT_1_R; however, replacement of Asp in AngII with sarcosine (N-methyl-glycine) not only restores bioactivity but increases the activity of agonist, antagonist, and inverse agonist analogues. A bend produced at the N-terminus by the introduction of the secondary amino acid sarcosine is thought to realign the functional groups that chaperone the C-terminal portion of AngII, allowing transfer of the negative charge originating at the C-terminus to be transferred to the Tyr hydroxyl-forming tyrosinate anion, which is required to activate the receptor and desensitizes the receptor (tachyphylaxis). Peptide (sarilesin) and nonpeptide (sartans) moieties, which are long-acting inverse agonists, appear to desensitize the receptor by a mechanism analogous to tachyphylaxis. Sartans/bisartans were found to bind to alpha adrenergic receptors resulting in structure-dependent desensitization or resensitization. These considerations have provided information on the mechanisms of receptor desensitization/tolerance and insights into possible avenues for treating addiction. In this regard sartans, which appear to cross the blood–brain barrier more readily than bisartans, are the preferred drug candidates.

## 1. Introduction

Substance addiction is clinically defined as a chronic, relapsing neuropsychiatric disorder characterized by a compulsive and overwhelming desire to seek and take harmful substances (e.g., alcohol, tobacco, illicit and prescription drugs) [1,2,3], despite adverse consequences, which results in psychological and/or physical dependence [4,5,6]. According to the United Nations World Drug Report 2019, approximately 35 million people globally suffer from drug use disorders while only 1 in 7 receive treatment for substance abuse [7,8]. Relapse rates after inpatient substance rehab are high: 40–60% within 3 months and 70–80% within 12 months [9]. New therapies are crucial for better outcomes.

The blood–brain barrier (BBB) is a selective membrane of brain endothelial cells facilitating communication among vascular cells, neuroglia, and neurons, regulating molecular exchange with the peripheral circulation [10,11,12,13]. The BBB is responsible for maintaining homeostatic balance by creating a tightly controlled microenvironment [14], allowing for (i) exchange of nutrients, hormones, ions (e.g., Ca^2+^, Cl^−^, K^+^ and Na^+^) and molecules between the blood and brain that are necessary for optimal synaptic and neuronal activity, (ii) removal of metabolites and waste products from the brain and (iii) protection and defense against infiltrating pathogen insult, endogenous and exogenous neurotoxins and other potentially dangerous substances (e.g., alcohol, cocaine, amphetamine and nicotine) that may consequently pass from circulation into the brain [11,15,16]. The neurobiological mechanisms underlying compulsive substance abuse and addictive potential remain relatively elusive [3,4,17,18]. Together with other influences, such as genetics, environment, behavior and social influences, development of an overall treatment approach to combat substance abuse is complicated [4,5,19,20,21]. Recently, the interaction(s) between the substance of abuse and the BBB has emerged as a potential therapeutic target for substance abuse-related neuropathology [22,23]. BBB dysfunction arises from structural weakening, neurotoxic molecule leakage, transporter dysfunction affecting nutrient supply, and neuronal damage due to metabolite build-up and inflammation [22,24,25]. Understanding these mechanisms is crucial for treating substance abuse.

**Figure 1:**
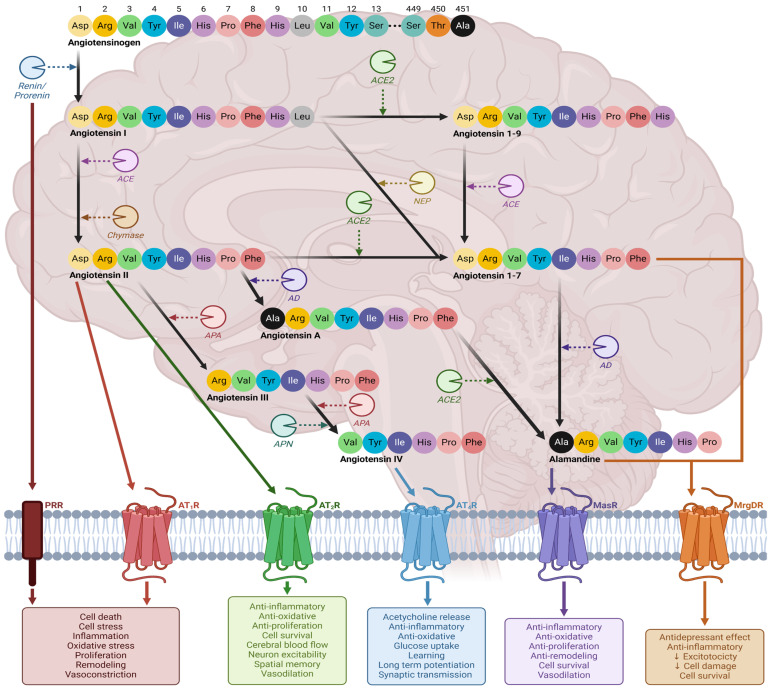
Schematic diagram of the brain RAS, depicting the various peptides, enzymes and receptors involved and the subsequent effect on specific neurological functions when activated by their ligands [44,45,46]. Angiotensinogen is first converted to AngI by renin and its precursor, prorenin. AngI can be metabolized to AngII by ACE and chymase, converted to Ang(1–9) by ACE2 or catalyzed into Ang(1–7) by neprilysin [31,45]. AngII can associate with either AT_1_R or AT_2_R, resulting in detrimental or homeostatic promoting effects, respectively [31,45]. Furthermore, AngII can then be converted to AngIII by aminopeptidase A (APA), AngA by aspartate decarboxylase (AD) or Ang(1–7) by ACE2 [31,45]. The resulting Ang III peptide is further acted upon by APA and alanyl aminopeptidase to become AngIV, which interacts with AT_4_R to promote neuroprotective effects [31,45]. Ang(1–9) can be catalyzed by ACE to either directly bind to the Mas-related G-coupled protein receptor (MrgDR) or can be acted upon by AD into alamandine, interacting with the Mas1 oncogene receptor (MasR) [31,45]. Finally, AngA can also be converted to alamandine by ACE2 [26]. Activation of prorenin receptor or AT_1_R by renin/prorenin and AngII, respectively, results in deleterious events, such as cell death, inflammation, oxidative stress, and vasoconstriction [44,45,46]. In contrast, activation of AT_2_R, AT_4_R, MasR and MrgDR induces opposing advantageous effects [44,45,46]. Abbreviations: ACE, angiotensin-converting enzyme; AD, aspartate decarboxylase; Ala, alanine; AngI, angiotensin; AngII, angiotensin II; AngIII, angiotensin III; AngIV, angiotensin IV; APA, aminopeptidase A; APN, alanyl aminopeptidase; Arg, arginine; Asp, aspartic acid; AT_1_R, angiotensin II type 1 receptor; AT_2_R, angiotensin II type 2 receptor; AT_4_R, angiotensin II type 4 receptor; His, histidine; Ile, isoleucine; Leu, leucine; MasR, Mas1 oncogene receptor; MrgDR, Mas-related G-coupled protein receptor; NEP, neprilysin; Phe, phenylalanine; Pro, proline; Ser, serine; PRR, prorenin receptor; RAS, renin–angiotensin system; Thr, threonine; Tyr, tyrosine; Val, valine. Figure made using Biorender.com (access date: 10 March 2024).

**Figure 2:**
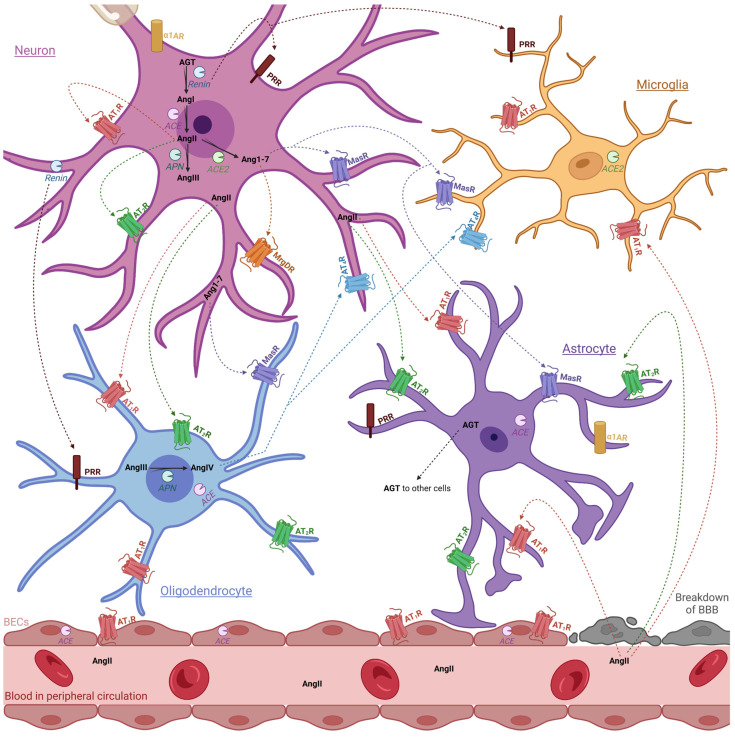
Expression profile of brain RAS components and α1 adrenergic receptors within the BBB, including astrocytes, microglia, neuron, and oligodendrocytes [33,47,48]. While it was once thought that AngII was unable to pass through the BBB due to its size and hydrophobic nature, there is evidence to the contrary. Firstly, in spontaneous hypertensive rats, breakdown of the BBB may increase permeability, allowing for extravasate access of AngII into the hypothalamus [49]; however further research is required to substantiate this claim in humans. Moreover, AT_1_R-mediated transcytosis of AngII by brain endothelial cells (BECs) of the BBB may allow entry of AngII to regulate autonomic function [50]. Abbreviations: ACE, angiotensin-converting enzyme; AGT, angiotensinogen; Ang, angiotensin; APA, aminopeptidase A; AT_1_R, angiotensin II type 1 receptor; AT_2_R, angiotensin II type 2 receptor; AT_4_R, angiotensin II type 4 receptor; α1AR, adrenergic receptors; BECs, brain endothelial cells; MasR, Mas1 oncogene receptor; MrgDR, Mas-related G-coupled protein receptor; PRR, prorenin receptor; RAS, renin–angiotensin system. Key: red dashed arrow = peptide binds to and activates AT_1_R; green dashed line = peptide binds to and activates AT_2_R; orange dashed arrow = peptide binds to MrgDR; purple dashed arrow = peptide binds to and activates MasR; brown dashed arrows = peptide binds to and activates PRR. Figure made using Biorender.com (access date: 10 March 2024).

The renin–angiotensin system (RAS) is a critical regulator of blood pressure by controlling blood volume, fluid and electrolyte balance and systemic vascular resistance through coordinated crosstalk between the heart, vasculature, and kidneys [26,27,28]. The RAS promotes hemodynamic and cardiovascular homeostasis through two opposing systems: the traditional and counterregulatory axes [29]. Importantly, the discovery of an independent RAS expressed locally in the cells of the nervous system (Figure 1 and Figure 2) has been shown to be involved in learning and memory consolidation, intellect and cognition, vasopressin secretion, appetite, and thirst [30,31] and may have an unknown role in maintaining BBB integrity [32,33]. Overactivation of the traditional axis (angiotensin-converting enzyme (ACE), angiotensin II (AngII) and angiotensin II type 1 receptor (AT_1_R)), leads to RAS dysfunction, triggering oxidative stress, neuroinflammation, deleterious remodeling and apoptosis. This is associated with neurodegenerative diseases like Alzheimer’s, Huntington’s, multiple sclerosis [34], Parkinson’s [35], cerebrovascular disease [36], cognitive impairment [37] and encephalopathy [38]. Correlations between brain RAS dysfunction, substance abuse and addiction, and drug seeking behavior have recently been reported in animals and humans [39]. Psychoactive substances disrupt brain RAS homeostasis by altering the expression of components involved in the traditional and counterregulatory axes. For example, nicotine upregulates AT_1_R and downregulates AT_2_R and ACE2 expression in neurons and glial cells [40,41], cocaine increases the expression and activity of ACE [42], amphetamine augments AT_1_R and AngII expression [43] and excessive alcohol consumption decreases mRNA levels of ACE2, angiotensin 1–7 (Ang(1–7)) and elevates ACE1 activity, Ang II expression and AT_1_R mRNA [39]. Thus, restoring the underlying brain RAS balance may be key to the treatment of substance abuse and addiction.

The octapeptide AngII (DRVYIHPF) plays a critical role in cardiovascular homeostasis and blood pressure regulation [26]. To combat cardiovascular pathologies (e.g., atherosclerosis, coronary artery disease and hypertension), important pharmaceuticals have been developed which either inhibit its synthesis (i.e., angiotensin-converting enzyme inhibitors) or block its receptor (i.e., AngII receptor blockers (ARBs)) [26]. Additionally, AngII acts on T cell receptors [51], modulating the immune and inflammatory responses that result in autoimmune diseases (e.g., rheumatoid arthritis, systemic lupus erythematosus and multiple sclerosis) [52], diseases arising from chronic hyper-inflammation (e.g., atherosclerosis, coronary artery disease and chronic kidney disease) [53,54] and neurodegenerative disorders (e.g., Alzheimer’s disease, multiple sclerosis and motor neuron disease) [55,56]. More recently, sartans (non-peptide ARBs) have proven effective in combating COVID-19, not only by inhibiting the infective process [57] but also by blocking AngII-mediated inflammatory responses (i.e., cytokine storm) [58]. Therefore, understanding the details of the interaction of AngII with its primary receptor, AT_1_R forms the basis for the development of the next generation of drugs.

The relationship between ARBs, addiction and the BBB has been reported in the literature. The ARB candesartan has been shown to reduce morphine-induced inflammatory response and cellular activation in murine-derived microglial cells [59], significantly decrease methamphetamine self-administration and seeking behavior in male Sprague–Dawley rats [60] and pretreatment with candesartan in Wistar rats has been shown to attenuate the development of amphetamine-induced behavior sensitization [61,62]. Moreover, acute intracerebroventricular administration of telmisartan, but not losartan, reduces alcohol consumption in Sardinian alcohol-preferring rats [63] and treatment with valsartan has been observed to prevent morphine-tolerance in Wistar rats [64]. The discrepancies observed between ARBs and their potential to be used in addiction may be due to their ability (e.g., azilsartan, candesartan, telmisartan and valsartan) or inability (e.g., eprosartan, irbesartan, losartan and olmesartan) to penetrate the BBB [37,65].

Additionally, the postsynaptic G-protein coupled receptor (GPCR) and alpha-1 (α1) adrenergic receptor, have also gained interest as potential therapeutic targets for the treatment of substance abuse [66,67,68]. α1 adrenergic receptors have an intimate relationship with the RAS, as binding of the neurotransmitter, norepinephrine, and the neurohormone, epinephrine, regulates sympathetic nervous system tone by mediating blood pressure through vasoconstriction of vascular smooth muscle cells (Figure 2) [69,70,71]. The α1 adrenergic receptor has been shown to play a role during cocaine and morphine abuse and addiction by modulating the release of neurotransmitters that are part of the reward system. For example, during cocaine and morphine exposure α1 adrenergic receptors participate in the enhancement of dopamine neuronal excitability while simultaneously modulating glutamatergic neurotransmission and reducing gamma-aminobutyric acid inhibition of dopamine neurons, resulting in drug seeking behaviors and sensitization [67,72]. Thus, a dual treatment, involving restoration of RAS and inhibition of the α1 adrenergic receptor, may provide novel treatment to combat substance abuse and addiction.

## 2. Results and Discussion

### 2.1. Sarmesin and Sarilesin

Pharmacological studies on smooth muscle tissues have shown that methylation of tyrosine (Tyr) hydroxyl leads to the competitive antagonist [Sar1Tyr(Me)4]AngII or sarmesin. Substituting the aromatic sidechain of phenylalanine (Phe)8 with an aliphatic residue, as seen in [Sar1Ile8]AngII or sarilesin, results in an inverse agonist. This also desensitizes the receptor to AngII, akin to tachyphylaxis [73]. Interestingly, methylation of the Tyr4 hydroxyl of sarilesin converts this peptide from a desensitizing (slowly reversing) inverse agonist into a reversible competitive antagonist, such as sarmesin, illustrating that the process of receptor desensitization is also dependent on tyrosinated anion formation, as with agonist activity. Sartans, like sarilesin, are long-acting inverse agonists with slowly reversing (desensitizing) effects. Their tyrosinate anion promotes tachyphylaxis/desensitization by AngII and sarilesin, mimicked by carboxylate or tetrazole in sartans.

### 2.2. The Role of Sar1 in AngII

All AngII analogues benefit from Sar1 presence, including AngII (enhanced to a superagonist), competitive antagonist sarmesin, and long-acting inverse agonist sarilesin. Replacement of Sar1 with natural Asp1 reduces sarmesin and sarilesin activities significantly, suggesting stabilization of the tripartite conformation by the charge relay system (CRS) interacting with the N-terminus; however, clearly in the case of sarmesin only a partial network (His–carboxylate) can form; so that the correct placement of Tyr(Me) by interaction with the N-terminal becomes even more essential. Indeed, nuclear magnetic resonance studies have revealed no substantive conformational differences among AngII, sarmesin and sarilesin, despite their diverse properties [74].

Previous studies have suggested that the positively charged guanidino cation of Arg acts as a chaperone, potentially stabilizing the tyrosinate anion [75]. Interaction between Arg guanidino cation and Asp carboxylate anion likely influences their positioning, with Asp carboxylate also interacting with the positively charged N-terminal amino group. This cluster of charged groups, held together by strong electrostatic interactions, likely profoundly influences the molecule’s structure. Further, studies have implicated a tripartite interaction in AngII, involving Tyr, histidine (His), and the C-terminal carboxylate [76,77]. This results in the transfer of charge from the C-terminus to form a tyrosinate anion, stabilized by interaction with the N-terminal part of the peptide, primarily the arginine guanidino group [76]. The significance of the N-terminal domain is demonstrated by the loss of bioactivity (< 5%) for Acetyl-AngII (loss of positive charge at N-terminus), for [Ala1]AngII or AngA (loss of negative charge on sidechain), and for [Ala2]AngII (loss of positive charge on sidechain). Thus, the cluster of three charged groups in the N-terminal region plays a significant role in maintaining the bioactive conformation of AngII. However, when the aspartic acid residue is replaced by sarcosine (Sar), there is an accompanying increase in bioactivity that can only be explained by a special realignment of the conformation of the N-terminal portion of the peptide, induced by a bend caused by the Sar residue. Bends/turns in the peptide backbone are induced by secondary amino acids (e.g., proline and Sar) and sterically hindered branched sidechain amino acids (e.g., valine and isoleucine). The presence of isoleucine and proline in the C-terminal domain of AngII creates a sharp gamma turn, facilitating tripartite interaction, while the presence of valine in the N-terminal region enables the arginine guanidino group to interact with the tyrosine sidechain.

### 2.3. Angiotensin A

**Figure 3:**
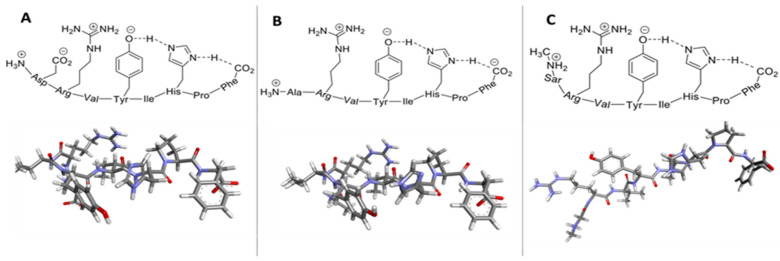
Two- and three-dimensional models depict intramolecular interactions of angiotensins. (**A**) The tyrosinate anion, formed by the charge relay system (CRS) (Tyr..His..CO_2_- of Phe) is stabilized by interaction with the arginine (Arg) guanidino cation, which is maintained in position by the aspartate (Asp) carboxylate anion, depicted bonded between two cations (resulting in bioactivity as charge is transferred to TyrOH). (**B**) Angiotensin A (AngA) alters the conformation of the N-terminal region, affecting the ability of the Arg cation to effectively chaperone the CRS (resulting in weak activity as charge transfer is only partial). (**C**) [Sar1] AngII creates a bend at the N-terminus restoring the position of the Arg guanidino cation, enabling it, together with the sarcosine (Sar) amino cation, to chaperone the CRS (enhancing bioactivity and functioning as a superagonist). Abbreviations: AngII, angiotensin II; C, carbon; H, hydrogen; His, histidine; Ile, isoleucine; N, nitrogen; O, oxygen; Phe, phenylalanine; Pro, proline; Tyr, tyrosine; Val, valine. Key: blue = nitrogen atoms; red = oxygen atom; grey= carbon atoms; white = hydrogen atoms.

The conformation of small peptides is largely driven by strong electrostatic interactions, especially ion pairing (salt bridge formation), which greatly influence conformational outcomes. In AngII, Asp carboxylate and Arg guanidinium side chain groups, together with the N- and C- terminals, are major energy determinants. Chemical reactivity studies on AngII suggest activation of the Tyr hydroxyl group, indicating interaction with the C-terminal carboxylate [77]. Additionally, His imidazole of AngII is activated, indicating interaction with the C-terminal carboxylate and TyrOH, possibly forming a CRS that links the TyrOH–His imidazole–carboxylate (of Phe). The CRS formation will be energetically favored because of the increased charge delocalization available if there is no extra strain produced in other parts of the molecule. Evidence from fluorescence lifetime spectroscopic studies [78] supports this mechanism. As a corollary of this, it is likely that the Arg guanidino cation would naturally move in to stabilize the Tyr anion formed by the CRS, acting as a chaperone. The loss of bioactivity (> 90%) associated with the substitution of Arg (e.g., with norleucine) supports the idea of an important role for the guanidino group. Activity is also greatly depreciated when the N-terminal amino cation is neutralized (e.g., by acetylation) or the Asp carboxylate anion is removed (e.g., by substitution with alanine (Ala), as in AngA. This suggests a complex interactive role for all three charged groups, possibly working in concert (Figure 3A–C). According to Figure 3A, in addition to the tripartite interaction in the C-terminal half of the AngII molecule, there is also a tripartite interaction in the N-terminal region invoking amino–carboxyl–guanidino, which presumably helps to position the guanidino to interact with TyrO-. The Asp carboxylate anion will naturally attempt to position itself to interact with both amino and guanidino cations. In Figure 3B, the removal of the Asp carboxylate disrupts this tripartite interaction such that the Arg guanidino is no longer close enough to stabilize TyrO- and there is a consequent loss of bioactivity (< 5%). Substituting Sar1 restores activity, implying that repulsion between amino and guanidino groups is compensated for by a turn in the backbone induced by Sar. This repositions the Arg sidechain group to chaperone TyrO- (Figure 3C) (considering the limitations imposed by this two-dimensional representation).

### 2.4. Receptor Interactions: Desensitization and Tachyphylaxis

**Figure 4:**
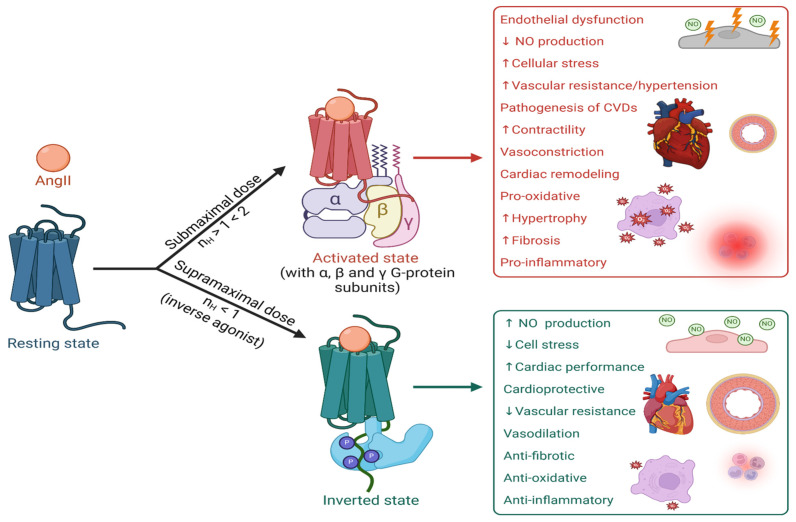
Receptor interactions of the cardiovascular effects of agonist and inverse agonist (desensitizing) effects at AT_1_R. The figure illustrates the dynamic interaction between AngII and AT_1_R under different conditions [82,83,84,85]. At baseline, AngII binds to AT_1_R in its resting state. At submaximal doses (n_H_ > 1 < 2), AngII forms an activated state with the alpha, beta, and gamma G-protein subunits, leading to endothelial dysfunction, the pathogenesis of cardiovascular diseases (CVD), vasoconstriction, cardiac remodeling, pro-oxidative stress, and inflammation. Conversely, at supramaximal doses (n_H_ < 1), inverse agonists induce an inverted state of the receptor. This results in an increase in nitric oxide (NO) and cardiac performance, decreased cellular stress, cardioprotection, vasodilation, anti-fibrotic, anti-oxidative, and anti-inflammatory effects conditions [82,83,84,85]. Desensitizing or inverse agonists form more stable salt bridges with residue R167 of AT1R, coupling the receptor to different signaling pathways and inducing long-term blockade, as seen with certain ARBs, like candesartan, telmisartan, and the peptide analogue sarilesin. Additionally, Figure 4 suggests a potential mechanism for tachyphylaxis, wherein high concentrations of agonists lead to receptor desensitization by forming stable salt bridges, resulting in a slow reversal process. This mechanism likely serves as a cellular defense mechanism against excessive cell stimulation, particularly in cardiovascular tissues, thereby protecting against the noxious effects of AngII. Abbreviations: P, phosphorylation. Key: ↑ = increases; ↓ = decreases. Figure created with Biorender.com (access date: 10 March 2024).

AngII interacts with the AT_1_R, leading to receptor dimerization and G-protein binding, leading to the contractile response [79]. The tyrosinate anion likely interacts with R167 or K199 of the receptor, creating a transient bond, which results in receptor activation [57]. Desensitizing/inverse agonists form a more stable salt bridge with R167 [80], coupling the receptor to different signaling pathways [81] and causing long-term blockade, particularly for certain ARB sartans (e.g., candesartan, telmisartan) and the peptide analogue sarilesin. This mechanism may also explain tachyphylaxis (Figure 4) where high agonist concentrations desensitize the receptor by forming a stable salt bridge, slowing reversal. This could serve as a cellular defense mechanism against overstimulation.

Studies on muscarinic partial agonists, which cannot produce the maximum response enabled by the natural agonist, are thought to have mixed agonist/inverse agonist properties, with inverse effects kicking in before the maximum response to the natural agonist can be achieved. Accordingly, all ligands, including the natural ligand, can produce concentration-dependent positive cooperativity (i.e., agonist activity) or negative cooperativity (i.e., inverse agonist activity) at receptors (Figure 4). Inverse agonism is associated with protracted desensitization effects, which may derive from the formation of more stable bonding with the receptor and/or second messenger [86]. Thus, tachyphylaxis, desensitization and inverse agonism represent interconnected facets of the same process. This phenomenon is influenced by both dosage and time, with the between doses impacting the cumulative degree of desensitization. Both factors also affect recovery time, or the time needed to re-sensitize the receptor and restore normal function. It is also possible that desensitization involves internalization of the receptors and that re-sensitization results from a delay in the shuttling of new receptors to the cell membrane.

### 2.5. Desensitization and Addiction

When a receptor undergoes desensitization, it develops tolerance to the ligand, necessitating higher doses for the same effect. This process can lead to dependence and withdrawal, hallmark symptoms of addiction. Consequently, receptor desensitization and addiction are interconnected phenomena, which can be investigated for therapeutic intervention. For example, in opiate addiction, methadone, a partial agonist methadone is used to mitigate opiate withdrawal symptoms by dampening both agonists and desensitization effects. Natural opiate agonists like endorphins/enkephalins are akin to fentanyl in potency, suggesting that many synthetic opiates (e.g., morphine, heroin and oxycodone) may act as partial agonists, with methadone being the weakest. Naloxone, a competitive antagonist, reverses the agonist effects without affecting opiate desensitization. To address addiction, we propose targeting the primary causal mechanism, receptor desensitization, potentially by dissociating it from the receptor. Without desensitization, tolerance, dependence, and withdrawal may be mitigated. However, this approach may limit the receptor’s responsiveness to pure agonists and competitive antagonists, potentially compromising its protective tachyphylactic response.

### 2.6. Therapeutic Intervention

One crucial aspect of desensitization is the duration it takes for the receptor to regain normal function (re-sensitization). Details of this time-dependence are poorly understood but may involve not only a stable salt bridge between ligand and receptor, as proposed for angiotensin, but also the dynamics of association/dissociation of the second messenger in the signaling pathway which invokes the inverse response. In the case of angiotensin, the inverse response is smooth muscle relaxation due to an unknown messenger, possibly a different G protein (Figure 4). In the context of opiate receptors, this reverse response could increase pain sensitivity, counteracting the analgesic effects of opiates. If the slow reacquisition of normal receptor function is the result of the slow dissociation of messenger(s) at or downstream of the receptor itself, it may be possible to decouple the receptor from this and inverting signaling pathway(s) without compromising the formation of the receptor dimer coupled to the G protein, which provides for agonist activity (Figure 4). High throughput screening for molecules selectively uncoupling the inverse agonist response could identify potential candidates. Therapeutic administration of such a “decoupler” molecule could simplify and predict withdrawal therapy titration, albeit with a heightened risk of overdose. Without the desensitization process, withdrawal could involve meticulously reducing agonist doses over time. This calibrated withdrawal approach may be applicable to various addictive substances. Individuals who are “endogenously compromised,” such as those with sub-physiological levels of agonist or compromised receptors (e.g., analogous to insulin receptor insensitivity in type II diabetes), could potentially be maintained on a prescribed dose of agonist plus decoupling agent. Removing the desensitization effects could reverse addiction while the addressing of any remaining psychological or “perceived need” aspects may require cognitive therapy.

### 2.7. Receptor Crosstalk and Compensatory Interactions

The majority of receptors, including GPCRs, are subject to a desensitization/tachyphylaxis mechanism, and have the potential to be decoupled from this desensitization pathway, possibly using a small molecular entity. For example, methamphetamine addiction, which is characterized by desensitization of the GPCR α1 adrenergic receptor, could perhaps be treatable by decoupling the signaling pathway, which leads to tachyphylaxis/desensitization. If there is crosstalk between receptors for different contractile ligands as a means of cardiovascular regulation, then the possibility exists that drugs like sartans, which principally desensitize AT_1_R could have an influence on α1 receptors. Indeed, in isolated smooth muscle assays, ARB sartans (e.g., candesartan, telmisartan) generally decrease the response of the α1 adrenergic receptor to phenylephrine, although notably the monovalent ARB sartan up-sensitizes the alpha receptor (implicating a subtle structure–activity relationship for this effect). Thus, deactivation of one GPCR can lead to upregulation or downregulation of a different GPCR. This compensatory mechanism could derive from allosteric effects between receptors bumping up against each other in the cell membrane, or more likely through interaction between their response transmission mechanisms. Apparently, receptors can be complex gatekeepers, suggesting that there may be common signaling pathways among different GPCR. We are in the process of examining the potential application of sartans for preventing the development of tolerance, and thereby addiction, to methamphetamine. In this context, there may possibly be a role for sartans in opiate addiction (also mediated by a GPCR).

### 2.8. Sartans Acting on Alpha Adrenergic Receptors

Agonist action at GPCR is associated with homotropic cooperativity (receptor dimerization) and heterotropic cooperativity (G-protein coupling), resulting in amplification of agonist affinity and responsiveness (Hill coefficient between 1 and 2). Antagonists can be either competitive antagonists (rightward shift of the response curve, Hill coefficient unchanged), or noncompetitive antagonists/inverse agonists. These demonstrate negative cooperativity (Hill coefficient < 1), couple to a different second messenger (possibly beta-arrestin) and desensitize the receptor for prolonged periods (possibly by invoking receptor internalization) (Figure 4). Many agonist ligands acting at GPCR become inverse agonists at higher concentration (tachyphylaxis). Agonist ligands for alpha adrenergic GPCR include epinephrine, phenylephrine and methamphetamine, and alpha blockers like prazosin are inverse agonists at alpha receptors.

**Figure 5:**
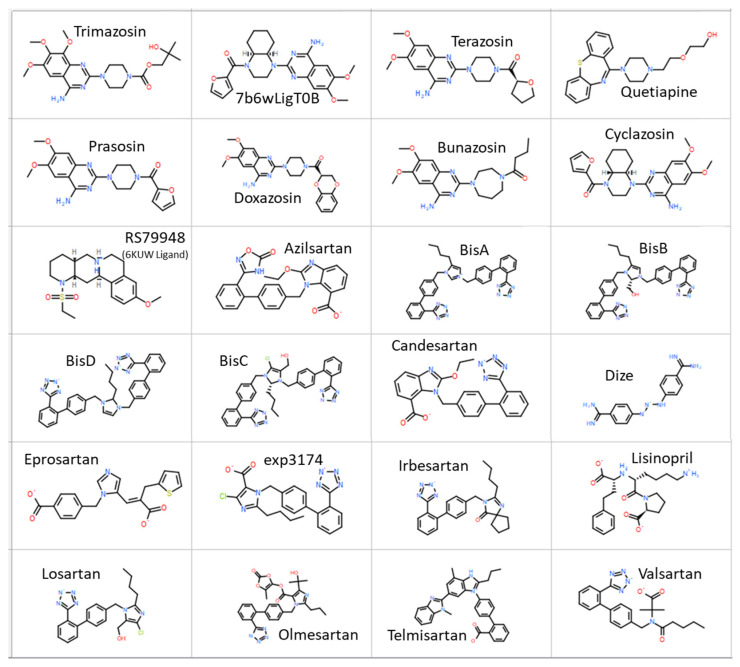
Structures of the ligands which were used for docking studies to the X-ray crystal structure of the alpha receptor are shown. Abbreviations: BisA, 4-Butyl-N,N0-bis([[20-(2H-tetrazol-5-yl)]biphenyl-4-yl]) methylimidazolium bromide; BisB, 4-Butyl-2-hydroxymethyl-N,N0-bis([20-(2H-tetrazol-5-yl)- biphenyl-4-yl])methyimidazolium bromide; BisC, 2-Butyl-4-chloro-5-hydroxymethyl-N,N0-bis([20-(2H-tetrazol- 5-yl)biphenyl-4-yl]methyl)imidazolium bromide; BisD, 2-Butyl-N,N0-bis([20-(2H-tetrazol-5-yl)biphenyl-4-yl]methyl) imidazolium bromide; Cl, chlorine; DIZE, diminazene aceturate; exp3174, losartan carboxylic acid; H, hydrogen; N, nitrogen; O, oxygen; S, sulfur. Key: green = chlorine; grey = hydrogen; blue = nitrogen; red = oxygen; yellow = sulfur.

**Figure 6:**
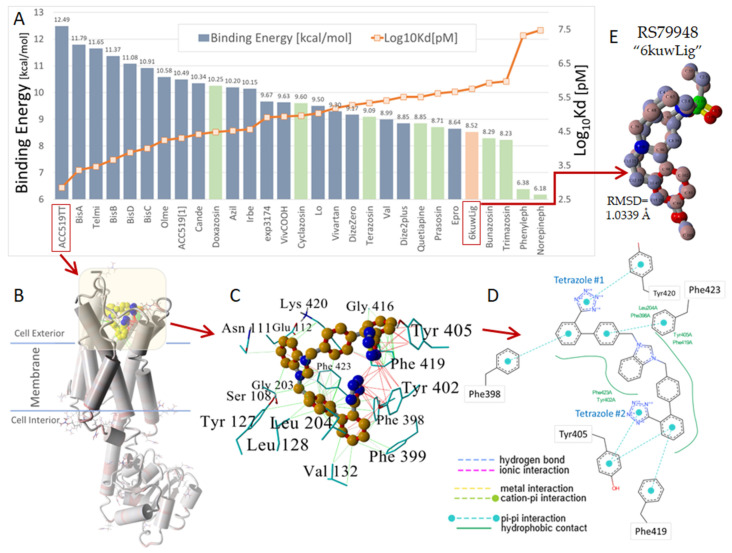
(**A**) Docking results obtained for 29 selected ligands, including various sartans (blue bars) and known inverse agonists to an alpha-2C-adrenergic receptor (green and orange bars), belonging to the GPCR class. Ligand docking was performed against the energy-minimized (AMBER14) alpha-2c-adrenergic receptor, PDB 6KUW, using AutoDock VINA with 900 runs per ligand with assigned AMBER14 atomic point charges and dihedral barriers [28]. Of the 29 ligands evaluated, the anionic bisartan ACC519TT exhibited the strongest docking energy (12.5 kcal/mol). ACC519TT outperformed the opioid-like ligand “6kuwLig” (compound RS79948:(8~(a)~(R),12~(a)~(S),13~(a)~(R))-12-orange bars) (accessed on 11 March, 2024) designed as a specific inhibitor of the 6KUW receptor. (**B**) Structure of the 6KUW opioid receptor with docked bisartan ACC519TT (yellow carbon atoms) embedded in the cell surface domain. The yellow shaded area approximates the docking region of interest. Residues shown in ethylsulfonyl-3-methoxy-5,6,8,8~(a),9,10,11,12~(a),13,13~(a)-decahydroisoquinolino [2,1-g][1,6]naphthyridine; https://www.rcsb.org/structure/6KUW; “stick” rendering indicate the locations of arginine residues for 6KUW. (**C**) Illustrates the 3D rendition of the docked ligand ACC519TT, while (**D**) presents the 2D interaction diagram calculated using PoseView-2D, ZBH-Center for Bioinformatics: https://proteins.plus/) (accessed on 11 March 2024). Principle binding interactions included non-covalent pi–pi resonances (red lines in (**C**) and cyan dashed lines in (**D**)) of both anionic tetrazole groups with proximal tyrosine residues (Tyr402 and Tyr405) and phenylalanine (Phe398). Hydrophobic interactions (green lines) were also heavily represented in ACC519TT binding. (**E**) Superposition of the PDB 6KUW X-ray crystallographic structure of the co-crystallized “6kuwLig” ligand (blue carbon atoms, as spheres) against docked ligand “6kuwLig” (maroon colored carbon atoms, as spheres). The superimposed molecules exhibited an RMSD of 1.0339 Angstroms, indicating that VINA algorithms were able to reproduce a high-quality experimental pose for this ligand. Abbreviations: ACC519TT, benzimidazole-N-biphenyl tetrazole; ACC519[1], benzimidazole-N-biphenyl tetrazole; Asn, asparagine; Azil, azilsartan; BisA, 4-Butyl-N,N0-bis([[20-(2H-tetrazol-5-yl)]biphenyl-4-yl]) methylimidazolium bromide; BisB, 4-Butyl-2-hydroxymethyl-N,N0-bis([20-(2H-tetrazol-5-yl)- biphenyl-4-yl])methyimidazolium bromide; BisC, 2-Butyl-4-chloro-5-hydroxymethyl-N,N0-bis([20-(2H-tetrazol- 5-yl)biphenyl-4-yl]methyl)imidazolium bromide; BisD, 2-Butyl-N,N0-bis([20-(2H-tetrazol-5-yl)biphenyl-4-yl]methyl) imidazolium bromide; Cande, candesartan; Dize, diminazene aceturate; Epro, eprosartan; exp3174, losartan carboxylic acid; Glu, glutamic acid; Gly, glycine; GPCRs, G-protein coupled receptors; Ireb, irbesartan; K_d_, dissociation constants; Leu, leucine; Lo, losartan; norepineph, norepinephrine; PDB, Protein Data Bank; Phe, phenylalanine; phenyleph, phenylephrine; RMSD, root mean standard deviation; Ser, serine; Telmi, telmisartan; Tyr, tyrosine; Val, valsartan (7A) or valine (7C); Å, angstrom.

**Figure 7:**
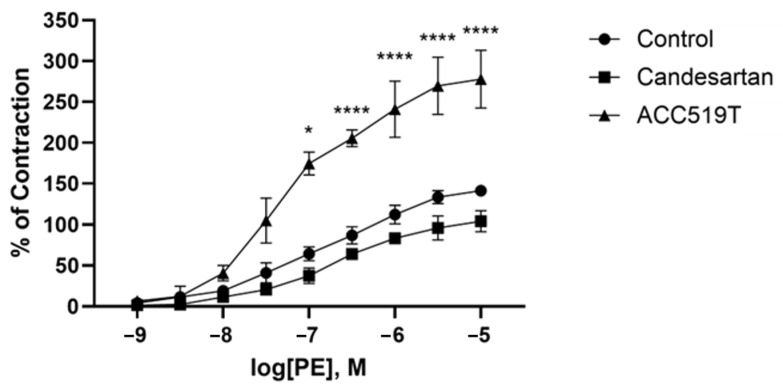
Effects of ACC519T and candesartan on phenylephrine-induced contraction of rabbit iliac arteries. Pretreatment with ACC519T significantly augmented contraction responses to phenylephrine (mean ± standard error of the mean (SEM)) (* *p* < 0.05, **** *p* < 0.0001); however, no differences in contraction abilities were observed in rings treated with candesartan (mean ± SEM). Abbreviations: ACC519, benzimidazole-*N*-biphenyl tetrazole; PE, phenylephrine.

**Figure 8:**
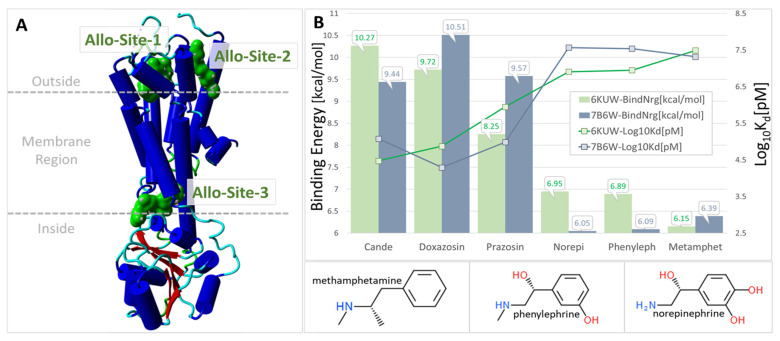
(**A**) 6KUW A chain showing predicted allosteric pockets (green shaded areas). Allosteric sites were calculated using Passer: Protein Allosteric Sites Server (https://passer.smu.edu/; access date: 5 March 2024). Docking was performed against allosteric site 1 for both 6KUW and 7B6W. (**B**) Binding energies of different drugs competed for the same site on the alpha receptors. Abbreviations: Allo, allosteric; Cande, candesartan; H, hydrogen; Kd, dissociation constants; Metamphet, methamphetamine; N, nitrogen; Norepi, norepinephrine; O, oxygen; Phenyleph, phenylephrine.

Computer simulations of the binding of ligands (Figure 5) to the alpha receptor (X-ray crystallographic structure) have been evaluated in terms of relative binding energies, as shown in Figure 6. Surprisingly, sartans/bisartans demonstrate greater affinity for the cell surface binding site than classical alpha blockers, and alpha agonists demonstrate the lowest affinity, which may be attributed to the absence of the cooperative effects of dimer and G protein to elevate affinity in these simulations. These findings explain the pharmacological ability of bisartans to reduce contraction in response to phenylalanine, an α1 adrenergic receptor agonist. We show that isolated iliac arteries from male New Zealand white rabbits, preincubated with benzimidazole-*N*-biphenyl tetrazole (ACC519T), had significantly augmented responses to a phenylephrine dose response, from 10^−7^ M (*p* < 0.05) to 10^−5^ M (*p* < 0.0001), when compared with untreated control rings (Figure 7). Moreover, rings pretreated with candesartan had no alteration in contraction responses when compared with controls (Figure 7). This suggests an ability to up-sensitize/re-sensitize the alpha receptor—as outlined above, this property has potential application in the treatment of addiction [21]. Computer simulations were also carried out on the alpha receptor dimer, as shown in Figure 8, and generally reflected the findings with the monomer. Despite their charge differences, all of the ligands shown in Figure 6 and Figure 8 competed for the same binding site on the alpha receptor and showed little tendency to bind elsewhere on the receptor molecule. Binding to alpha adrenergic receptors appears to be much more permissive than the strict rules for binding to angiotensin receptors outlined at the beginning of this treatise.

### 2.9. Perspectives of Sartans and Bisartans as Therapeutics by Blocking Arginine-Mediated Disease

Sartans, and in particular bisartans, discovered in our laboratories [87] have shown to be effective blockers of arginine, the dominant residue in promoting the cleavage of viral glycoproteins and activating severe acute respiratory syndrome coronavirus 2019 (SARS-CoV-2) viral infection [57,88]. The cleavage of SARS-CoV-2 spike protein by furin at the arginine rich multi basic sites, subunits (S)1/S2 (680-SPRRARS-686) and S2′ (810-SKPSKRS-816), occurs between the same R-S residues location in both sites, initiating SARS-CoV-2 infection. Therefore, arginine blockers (e.g., ARBs, sartans and bisartans) containing anionic tetrazole and/or carboxyl functional groups or dual tetrazoles with increased acidity represent promising repurposed antiviral drugs [54]. Thus, drugs in the sartan family can block furin activity and subsequent infection by obstructing basic amino acids at cleavage sites [57,88,89]. Clinical studies have shown the beneficial effect of ARBs, such as telmisartan and candesartan, in hypertensive patients infected with SARS-CoV-2 compared with patients not taking ARBs [90,91,92,93]. The interaction of sartans with the angiotensin-converting enzyme-2/receptor binding domain complex by computational and enzyme studies and the role of tetrazole functionality has been recently reported [94].

Bisartans bearing two symmetric biphenyl tetrazole warhead groups attached to the two nitrogens of the histidine ring, as in the BisA, BisB, BisC and BisD chemical structures, are unique and bind stronger when compared with other sartans bearing only one biphenyl tetrazole or carboxyl group [57]. Computational and enzymatic models demonstrate increased binding if the biphenyl tetrazole or carboxyl group is attached to the Benzimidazole ring and this is attributed to the extended pi electron resonance cloud inducing strong pi–pi interactions between ligand and receptor [57,94]. Additionally, bisartans have shown extraordinary properties in producing biological effects with AT_1_R, even at ultra-high dilutions, a phenomenon interpreted by entangled quantum pharmacology mechanism [95].

In summary, arginine plays a crucial role in the ligand–protein interactions across several biological systems, by stabilizing conformations and strongly binding to anionic sites [96,97]. Anionic sartans, particularly bisartans, exhibit potent binding capabilities, effectively neutralizing cationic arginines. This mechanism holds significance in protecting the biological systems from disease processes, such as those mediated by arginines, as in the cleavage of the SARS-CoV-2 spike protein by furin [98,99,100,101,102,103]. Utilizing additional synthetic and physicochemical methods could help resolve and confirm predicted computational interactions involving arginine, especially in microwave solid phase peptide synthesis. Tetrazole-containing compounds like sartans and bisartans, which block arginine have been investigated [57,58,76,104,105].

### 2.10. Sartans and Bisartans and the Blood–Brain Barrier

Although sartans are anticipated to readily cross the BBB, the zwitterion nature of bisartans makes it less likely that they will penetrate the BBB and thereby gain access to brain angiotensin receptors. Accordingly, central effects of ARBs due to actions at brain receptors should be more pronounced for sartans than bisartans. As it turns out, whereas bisartans are often more potent than sartans in isolated smooth muscle assays, bisartans tend to be less potent in pressor assays [21], where a component of blood pressure regulation is likely to be centrally mediated. Thus, any differences in biological activity between sartans and bisartans, when administered intravenously, can be anticipated to derive from central brain effects. Therefore, when considering processes related to addiction, which are brain-centric, sartans can be expected to be more useful therapeutic entities than bisartans.

## 3. Materials and Methods

### 3.1. In Silico Studies

Molecular simulation studies of our compounds into the protein targets were carried out using the open-source program Autodock 4.0 included in Auto-Dock Tools 1.5.6. For this study, the crystal structures of the proteins were extracted by the Protein Data Bank. Docking was performed using non-periodic (walled) boundaries that effectively confined ligands to an approximately 17 × 17 × 32 Å cuboid volume. The best hits and ligand conformational poses are expressed as kcal/mol free energy of binding.

Virtual ligand screening (VLS) was performed using AutoDock VINA [106,107] with default parameters. Receptor preparation, including designation of the docking region of interest (i.e., docking cell boundaries), hydrogen bond optimization, rebuilding of residue sidechains with missing atoms, addition of missing backbone atoms, deletion of water molecules that overlap with other atoms, correction of bond orders, etc., was undertaken with Yet Another Scientific Artificial Reality Application (YASARA) Dynamics molecular modeling software (YASAR Biosciences GmbH, http://www.yasara.org/) (accessed on 10 March 2024) [108]. Three-dimensional ligand structures were prepared and optimized in YASARA or Hyperchem (http://hypercubeusa.com/) (access on 10 March 2024) and saved in SDF format. For each ligand, the best hit from 900 runs was recorded and expressed in kcal/mol free energy of binding. Dissociation constants (K_d_) expressed in pM units were calculated from the binding energies. VINA uses a fast grid-based search method, and grids are created inside the walled docking cell, so atoms outside the cell do not influence the grid and thus the docking result. Note that in this report more positive docking scores correspond with stronger ligand–receptor interactions.

### 3.2. Ex Vivo Studies

#### 3.2.1. Animal Model, Ethics Approval and Humane Dispatchment

Male New Zealand White rabbits (*n* = 3) at 8–10 weeks of age were purchased from Flinders City University (Adelaide, SA, Australia) and were housed at Victoria University, Werribee Campus Animal Facilities, VIC Australia. Animals were kept on a 12 h day/night cycle, maintained at a constant temperature of 21 °C and relative humidity level between 40 and 70% and were aged until 16 weeks. Rabbits were fed normal chow diet pellets (Specialty Feeds, Glen Forrest, WA, Australia) and food and water were supplied ad libitum. All experimental procedures were conducted in accordance with the National Health and Medical Research Council ‘Australia Code of Practice for the Care and Use of Animals for Scientific Purposes’ (8th edition), 2013; https://www.nhmrc.gov.au/about-us/publications/australian-code-care-and-use-animals-scientific-purposes (accessed on 22 February 2024) and was approved by the Victoria University Animal Ethics Committee (VUAEC#17/013).

Rabbits were first sedated using a subcutaneous injection of medetomidine (0.25 mg/kg) at the ‘scruff’ or base of the neck to reduce stress and anesthetized using the inhalant isoflurane (4%). When loss of corneal and palpebral pain reflex was observed, an incision was made at the lower abdomen and the subcutaneous tissue and muscles were dissected to expose the inferior vena cava. Rabbits were humanely dispatched by inferior vena cava exsanguination and death was signified by dissection of the diaphragm. A T-tube was introduced distal to the aortic arch to allow adequate flushing of the aorta, aortic bifurcation, and iliac arteries with cold (4 °C) oxygenated Krebs–Henseleit (Krebs) (118 mM, NaCl; 4.7 mM KCl; 1.2 mM MgSO_4_·7H_2_O; 1.2 mM KH_2_PO_4_; 25 mM NaHCO_3_; 11.7 mM glucose; and 1.25 mM CaCl_2_) (pH: 7.4). The left and right iliac arteries were retrieved from each animal and, under a light microscope, were cleaned of connective and adipose tissue and cut into 2 mm rings for isometric tension myography studies.

#### 3.2.2. Drug Incubations and Isometric Tension Myography Studies

Iliac artery rings were immediately and sequentially placed into adjacent organ baths (OB16, Zultek Engineering, Melbourne, VIC, Australia) filled with 5 mL of Krebs and were acclimatized for 30 min. To replicate a physiologically relevant environment, baths were maintained at 37 °C and continuously bubbled with carbogen (95% O_2_/5% CO_2_). After acclimation, rings were mounted between two metal organ hooks attached to force displacement transducers, stretched to 0.5 g, and equilibrated for a further 30 min. Rings were then refreshed, re-stretched, and again equilibrated for 30 min before drug incubations. To investigate the effects of candesartan (a commercially available and commonly prescribed ARB) (Cat#9003239, Cayman Chemical, Ann Arbor, MI, USA) and ACC519 (an ARB that has been newly synthesized by our laboratory), rings serving as part of control groups were not incubated with any drug and were left to rest for 10 min, while the ARB experimental group rings were incubated with ACC519T (10^−6^ M) or candesartan (10^−6^ M) for 10 min. A phenylephrine (selective alpha-1 adrenergic receptor agonist) (Cat#P6126, Sigma Aldrich, St. Louis, MO, USA) dose response (10^−9^ M–10^−5^ M; each dose added at 2 min intervals) was then performed to determine the ability of drugs to alter contraction responses. Following the completion of dose–response studies, rings were refreshed, allowed to return to baseline tension, and contracted with high potassium physiological solution (125 mM) (125 mM/L KCl; 1.2 mM/L MgSO_4_·7H_2_O; 1.2 mM/L KH_2_PO_4_; 25 mM/L NaHCO_3_; and 11.7 mM/L glucose; and 1.25 mM CaCl_2_) (pH: 7.4) to determine maximal standard contraction responses.

#### 3.2.3. Statistical Analysis

GraphPad prism (version 10.2.0) was used for the statistical analysis of isometric tension studies using a two-way ANOVA, followed by Sidak’s post hoc test to determine significance. The significant *p*-value was set at *p* < 0.05, and all data are represented as the mean ± standard error of mean (SEM).

## 4. Conclusion and Future Prospects

Research on angiotensin has shed light on the intricate relationship between receptor desensitization and peptide structure. It has been observed that, at elevated concentrations, agonists shift their behavior, becoming inverse agonists due to altered binding modes with the receptor. This phenomenon, alongside tachyphylaxis and desensitization, forms a complex interplay that leads to the development of tolerance, physical dependence, addiction, and eventual withdrawal symptoms. Understanding the structural determinants underlying receptor desensitization and inverse agonism, especially in the context of high agonist concentrations, is paramount. This knowledge can significantly contribute to the development of effective strategies aimed at preventing addiction and managing withdrawal symptoms associated with physical dependence on agonists. Additionally, exploring the intricate relationship between peptide structure, receptor desensitization, tolerance, addiction, and withdrawal can unearth valuable insights. These insights may lead to the identification of novel therapeutic targets for intervention and provide new avenues for addiction treatment. Cross-tachyphylaxis observed between angiotensin receptors and α1 adrenergic receptors highlights the interconnectedness of GPCRs. This phenomenon suggests potential opportunities for pharmacological intervention in addiction treatment by targeting shared pathways. Among the potential treatment options, drugs targeting angiotensin receptors, particularly ARBs, like sartans, present a promising avenue for addressing methamphetamine addiction. This is due to their ability to penetrate the blood–brain barrier and their beneficial effects on diseases associated with dysregulation of RAS.

Sartans, known for their superior BBB permeability compared with bisartans, emerge as preferred candidates for treating amphetamine addiction. ACC519, a sartan exhibiting potential for re-sensitizing α1 adrenergic receptors and possibly opioid receptors, holds significant promise in this regard. Given these promising avenues, further research into the pharmacological properties and therapeutic potential of sartans and related compounds in addiction treatment is warranted. However, to bridge the gap between research findings and real-world applications in addiction treatment, further steps could involve clinical trials by which to assess the efficacy and safety of utilizing sartans or related compounds as potential therapeutic interventions. These trials could compare the outcomes of sartan-based treatments with existing addiction treatments, evaluating factors such as withdrawal symptoms, relapse rates, and overall patient wellbeing. Additionally, conducting in vivo studies using animal models of addiction could provide valuable insights into the mechanisms underlying sartan-mediated effects on addiction-related behaviors, helping to validate the proposed therapeutic mechanisms observed herein. This comprehensive approach would not only enhance our understanding of sartans’ potential in addiction treatment but also provide essential evidence for their practical application in clinical settings. This research could pave the way for the development of innovative and effective therapies to combat addiction and its associated complications.

## Data Availability

The data are not publicly available due to their commercial value. Interested parties can email corresponding authors.
